# Identification of a Novel Placental Peptide with Skin Reparative Properties by Cell-Surface Affinity Screening

**DOI:** 10.3390/ijms27198617

**Published:** 2026-09-26

**Authors:** Fangyu Xing, Jianping Gao, Zhenhu Guo, Yingjun Kong, Guifeng Zhang

**Affiliations:** 1 State Key Laboratory of Biopharmaceutical Preparation and Delivery, Institute of Process Engineering, Chinese Academy of Sciences, Beijing 100190, China; xingfangyu21@ipe.ac.cn (F.X.);; 2 School of Chemical Engineering, University of Chinese Academy of Sciences, Beijing 100049, China

**Keywords:** placental peptide, cell-surface affinity screening, dermal fibroblasts, microvascular endothelial cells, wound healing

## Abstract

Placental extracts are rich in bioactive molecules and exhibit broad reparative properties, yet the molecular constituents responsible for their effects on skin tissue repair remain insufficiently characterized. In this study, we employed a cell-surface affinity-based screening approach using dermal fibroblasts (BJ) and microvascular endothelial (HMEC-1) cells as dual biological baits to capture bioactive peptides directly from placental extracts. Through liquid chromatography–tandem mass spectrometry (LC-MS/MS) analysis and background subtraction, a peptide with dual-cell targeting potential, GPPVSE, was identified. In vitro, GPPVSE significantly promoted the viability, proliferation and migration of both BJ and HMEC-1 cells and enhanced endothelial tube formation. In vivo, topical 20 μM GPPVSE in a mouse full-thickness skin defect model achieved an 84.7% wound closure rate by day 8, outperforming both phosphate-buffered saline (PBS) and epidermal growth factor (EGF) groups. Histological analysis revealed that GPPVSE promoted granulation tissue maturation, strengthened epidermal–dermal junction integrity, and enhanced regeneration of skin appendages, including hair follicles and sebaceous glands, during the remodeling phase. Collectively, GPPVSE provides valuable molecular insights into the reparative activity of placental extracts and establishes a potentially adaptable cell-based paradigm for mining functional peptides from complex natural sources.

## 1. Introduction

The placenta, a transient organ unique to mammalian pregnancy, plays an essential role in fetal development and protection [1]. Postpartum placental tissues have been developed into various derivatives, such as placenta-derived stem cells, umbilical cord blood serum, placental conditioned medium, placental extracts, and placental decellularized extracellular matrix, which have wide applications in medicine, biomedical engineering, and other fields [1,2,3,4]. Notably, placental extracts have attracted great attention, benefiting from their anti-inflammatory, antioxidant, and immunomodulatory properties, as well as their capacity to regulate cellular behavior [5,6,7,8]. Generally speaking, the preparation of placental extracts involves a rigorous process including tissue collection, washing, homogenization, extraction, filtration, purification, and sterilization [9,10]. The obtained product comprises a rich repertoire of bioactive molecules, such as proteins, peptides, amino acids, vitamins, and nucleic acids [9,10,11], This rich composition and its superior biological properties drive the clinical use of placental extracts in liver dysfunction, osteoarthritis, and wound repair [2,12,13]. But the specific relationship between the complex molecular composition of placental extracts and their exact biological effects has yet to be fully elucidated.

Among various applications, the potential of placental extracts in wound repair has received increasing attention [2,10]. Previous studies have shown that placental extracts upregulate the expression of extracellular matrix (ECM) genes in human dermal fibroblasts [14]. Furthermore, they accelerate wound healing by stimulating fibroblast proliferation and increasing the expression of TGF-β1 and FGF at the wound tissue [15]. Additionally, placental extracts exhibit remarkable pro-angiogenic properties across various models, including endothelial tube formation and rat aortic ring assays [16]. Moreover, they have been shown to enhance endothelial cell viability, proliferation, and migration under high-glucose conditions by restoring PI3K/Akt/ERK1/2 signaling pathways and inhibiting apoptosis [17]. These findings strongly suggest the presence of specific bioactive components within placental extracts capable of exerting a dual regulatory effect on both dermal fibroblasts and endothelial cells. However, the identification and capture of specific bioactive components is difficult due to the complexity of placental extracts. Traditional isolation techniques primarily rely on physicochemical properties, such as molecular weight or polarity, to separate components without an identification function for specific bioactive components [18,19,20]. Therefore, an efficient, target-driven peptide screening strategy is urgently required to capture and identify the specific bioactive components responsible for mediating dual regulatory effects on both dermal fibroblasts and endothelial cells.

It is well established that the binding of extracellular molecules to cell membrane receptors triggers cellular phenotypic changes and downstream signaling cascades [21,22,23,24,25,26]. For example, the specific binding of CADM4 to its target receptor (CADM1) on adult T-cell leukemia (ATL) cells induces morphological spreading and alters cellular adhesion behavior [23]. To investigate such interactions, cell membrane proteins have been successfully utilized as affinity baits to capture novel interacting partners [27,28,29]. For instance, by incubating Plasmodium-derived peptide probes with human erythrocyte membrane extracts, researchers identified peptide targeted membrane receptors via mass spectrometry [28]. This strategy confirm that peptide fragments can effectively bind native membrane proteins independently of full-length structures. To further preserve the native conformation of membrane proteins, screening strategies have evolved to use whole live cells as affinity probes [30,31,32]. By overexpressing target membrane receptors on live cells, researchers have successfully screened and captured specific binding ligands, proving that live cell surfaces can serve as highly efficient in situ probes to capture unknown binding molecules [32]. Taken together, bioactive peptides function by specifically recognizing target cell receptors, and intact live cells effectively provide the optimal native conformation required for this recognition. Therefore, employing a live-cell affinity bait strategy to target, capture, and identify functional peptide components from placental extracts is highly feasible from both theoretical and methodological perspectives.

In this study, we successfully identified a novel peptide, GPPVSE, with dual-cell targeting potential, by cell-surface affinity screening which utilizes dermal fibroblasts and microvascular endothelial cells as natural biological baits to enrich cell-associated peptides. Subsequently, the synthesized peptide was evaluated both in vitro and in vivo. In vitro functional assays demonstrated its capacity to promote fibroblast proliferation and migration, as well as endothelial cell proliferation, migration, and tube formation. The GPPVSE was further evaluated in an animal model of skin wound repair and demonstrated efficacy in accelerating wound closure in vivo. By pinpointing this specific bioactive sequence, this work provides a compelling molecular rationale underpinning the efficacy of placental extracts in skin repair. And we hope this target-driven peptide screening strategy using living cells can be further applied to identify more functional peptides for biomedical applications.

## 2. Results

### 2.1. Identification of Cell-Associated Peptides from Placental Extracts

A cell-surface affinity screening strategy was implemented using BJ and HMEC-1 cells as biological baits. Briefly, placental extracts were independently incubated with the two cell lines. Following incubation, extensive washing steps with PBS were performed to rigorously eliminate unbound or non-specifically adsorbed molecules. The specific peptides bound to the cell surface were subsequently eluted for LC-MS/MS identification (Figure 1a). The LC-MS/MS data derived from the placental extract and the cell-surface affinity eluates of BJ and HMEC-1 were processed to generate their respective peptide datasets. Overall, 236 peptides were identified in the placental extract, with the top 20 most abundant peptides detailed in Table 1. To reduce non-specific background signals, peptides detected in the PBS blank control were filtered out from the respective cell-associated peptide datasets. Intersection analysis of the placental extract dataset and the two cell-bound peptide datasets identified the target peptide, GPPVSE. The peptide was detected in the original placental extract and in the affinity eluates from both BJ and HMEC-1 cells. The peptide was identified with high confidence based on accurate precursor mass, consistent retention time, and peptide-spectrum match scores (Table 2). Its identification was supported by mass spectrum (Figure 1b–d) and MS/MS spectrum (Figure 1e–g) obtained from the placental extract, BJ cell eluate, and HMEC-1 cell eluate. Extracted ion chromatogram analysis (Figure 1h) further showed that the peptide was consistently recovered across three independent affinity elution replicates. These results indicated that the peptide effectively associates with both dermal fibroblasts and microvascular endothelial cells, supporting the validity of the affinity screening strategy.

To evaluate whether the biological function of peptide GPPVSE had been previously reported, literature and database searches were performed using PubMed, Web of Science, Google Scholar, and UniProt. Search terms included the full peptide sequence “GPPVSE”, the precursor protein name, and combinations with keywords such as “placenta”, “fibroblast”, “endothelial cell”, “angiogenesis”, “wound healing”, “cell migration”, and “bioactive peptide”. Searches were last performed on 11 May 2026. The peptide GPPVSE was mapped to histone H1.4 and histone H1.5 according to UniProt annotation. The identified sequence corresponds to residues 37–42 of the H1.4 precursor, and residues 40–45 of the H1.5 precursor. Given that this six-amino-acid sequence is 100% conserved across both H1.4 and H1.5 precursors, current MS data cannot definitively discriminate its singular protein origin. To the best of our knowledge, no prior studies have described the pro-reparative, pro-migratory, or pro-angiogenic properties of this peptide sequence. For subsequent functional validations, the GPPVSE peptide was commercially synthesized, and its purity (>98%), molecular weight, and MS/MS fragmentation were confirmed by HPLC and MS (Figure S1).

### 2.2. The Effect of Placental Peptide on the Viability, Proliferation and Migration of Fibroblasts In Vitro

Cell-surface binding may be associated with downstream cellular responses. We examined whether the identified peptide affected viability, fibroblast proliferation and migration. CCK-8 assays were performed to evaluate the effect of 24 h peptide treatment on BJ cells. The peptide exhibited excellent cytocompatibility, and no apparent cytotoxicity was observed across the tested concentration range of 2–500 μM. Within this wide safety window, the peptide exhibited a characteristic bell-shaped dose-response curve regarding cell viability (Figure 2a). The most pronounced viability-enhancing effect was achieved at 20 μM, which significantly elevated cell viability by 41.18% compared to the untreated control. At higher concentrations, this efficacy gradually diminished, with cell viability returning close to the baseline level. To further confirm whether this enhanced viability translated into actual cell division, we quantified viable cell numbers using a Trypan blue-staining assay. Consistent with the CCK-8 results, direct cell counting revealed that peptide treatment for 24 h significantly promoted fibroblast proliferation (Figure 2b). Quantitative analysis showed that the relative cell proliferation for the 2, 20, and 200 μM peptide groups increased by 17.98%, 26.44%, and 15.78% compared to the untreated control, respectively. The 20 μM group exhibited the most robust proliferative response, perfectly mirroring the peak efficacy observed in the cell viability assay. In the scratch assay, microscopic observation and quantitative analysis showed that the peptide significantly accelerated the migration of fibroblasts into the scratched area (Figure 2c,d). During the early phase of treatment (16 h, Figure 2d), the 20 and 200 μM groups showed greater wound closure than the control group. At 40 h (Figure 2d), the scratch closure rates for the 2, 20, and 200 μM peptide groups reached 88.60%, 89.73%, and 94.88%, respectively. Compared to the basal closure rate of 79.01% in the control group, these treatments yielded significant relative improvements in healing efficiency by 12.14%, 13.57%, and 20.09%, respectively. Because the assay was strictly conducted under serum-starvation conditions to arrest cell division, this accelerated closure reflects a genuine enhancement of cellular migration capacity, independent of the peptide’s pro-proliferative effects. Together, these findings indicate that the peptide promoted fibroblast proliferation and migration in vitro.

### 2.3. The Effect of Placental Peptide on the Viability, Proliferation, Migration and Pro-Angiogenic Activity of Microvascular Endothelial Cells In Vitro

Affinity screening showed that the identified peptide also bound to HMEC-1 cells. Therefore, we evaluated its effects on microvascular endothelial cell viability, proliferation, migration, and tube formation. CCK-8 assays showed that the peptide increased HMEC-1 cell viability after 24 h treatment. The 20 and 100 μM groups showed significant increases of 27.59% and 29.67%, respectively, relative to the control group (Figure 3a). Trypan blue viable cell counting further confirmed the peptide’s pro-proliferative effect (Figure 3b). Quantitative analysis showed that the relative cell proliferation for the 2, 20, and 200 μM peptide groups increased by 24.53%, 31.03%, and 25.43% compared to the untreated control, respectively. Consistent with the viability assay, all tested concentrations elevated the viable HMEC-1 cell count, with the 20 μM group exhibiting the maximum proliferative response. In the Transwell migration assay, the peptide increased HMEC-1 migration across the porous membrane (Figure 3c). Quantitative analysis (Figure 3d) revealed that treatment with 2, 20, and 200 μM increased the number of migrated cells by 1.42-, 1.80-, and 1.43-fold, respectively, compared with the control group. Matrigel tube formation assay was used to evaluate angiogenic behavior in vitro. After 8 h of culture, the peptide-free cells formed a basal capillary-like network. Peptide-treated cells formed denser networks with more interconnected structures (Figure 3e). Quantitative analysis (Figure 3f,g) confirmed these morphological observations. For total vessel length, the 2 μM group yielded no significant change, whereas both the 20 and 200 μM groups increased significantly in vessel length, reaching 1.14-fold of the control group. Junction formation was enhanced by 1.30- and 1.28-fold at 20 and 200 μM, respectively. Overall, the 20 μM concentration showed the most consistent effect across proliferation, migration, and tube formation assays. These results suggested that the peptide could promote endothelial cell proliferation, migration, and angiogenic network formation in vitro.

### 2.4. The Effect of Placental Peptide in Wound Healing

Given the promising in vitro capacity to promote the viability, proliferation, migration, and tube formation of fibroblasts and microvascular endothelial cells, we further evaluated the peptide for in vivo wound healing efficacy using a mouse full-thickness skin defect model. Four experimental groups were established, including a blank control (PBS) group, a positive control (20 μM EGF) group, and two peptide treatment groups at different concentrations (20 μM and 200 μM). Representative images of the wounds across all groups from days 0 to 12 are presented in Figure 4a,b. Macroscopically, compared to the blank control group, all treatment groups exhibited a pronounced reduction in wound area on day 4. By day 8, the 20 μM peptide group achieved the highest wound closure rate, approximately 84.7% (Figure 4c). By the end of the observation period, wounds in all groups were largely healed, with the residual wound areas in the 20 μM and 200 μM peptide groups further reduced to 11.4% and 7.2%, respectively (Figure 4c).

To further assess the repair quality at the histological level, H&E staining was performed on the wound tissues harvested on days 8 and 12. The H&E images from day 8 revealed distinct morphological differences during the mid-stage of healing (Figure 4d). Complete closure of the neo-epithelium was achieved in both the EGF group and the two peptide treatment groups. In contrast, the neo-epithelium in the PBS group was still migrating towards the wound center and had not yet bridged the defect. Notably, although the epithelium in the EGF group had closed, distinct gaps remained beneath the neo-epithelium, reflecting poor tissue integration. In contrast, the granulation tissue in the 20 μM peptide group was denser and tightly integrated with the overlying epithelium, indicative of more mature tissue remodeling. To objectively substantiate these morphological observations, quantitative analysis of the neo-epithelial thickness was performed on representative tissue sections (Figure 4e). Within the evaluated sections, the 20 μM peptide group demonstrated a significantly thicker and highly uniform neo-epithelium compared to the PBS group (*p* < 0.05). Furthermore, the epithelial layer in the 20 μM group was demonstrably better developed than that of the EGF group, indicating superior local epidermal regeneration. Furthermore, pronounced infiltration of eosinophilic inflammatory cells was observed in the high-concentration (200 μM) group, suggesting that an excessively high local peptide concentration might induce or prolong the inflammatory phase of wound healing [33]. The H&E images from day 12 (Figure 4f) demonstrated that the 20 μM peptide group exhibited a marked advantage in dermal remodeling, as evidenced by the abundant regeneration of skin appendages (such as hair follicles and sebaceous glands). In contrast, markedly fewer skin appendages were sparse in PBS and EGF groups. Taken together, these results demonstrated that the placental peptide at an optimal concentration (20 μM) not only significantly accelerated wound closure but also facilitated high-quality skin regeneration by promoting granulation tissue maturation, strengthening the dermal–epidermal junction, and inducing skin appendage regeneration.

## 3. Discussion

As a time-honored natural biologic, placental extracts have long demonstrated considerable clinical utility in skin repair, anti-aging, and tissue regeneration [1,4]. However, owing to their highly complex composition, the specific active molecules responsible for reparative effects have yet to be precisely identified [9]. Conventional screening approaches often rely on iterative column chromatography combined with in vitro activity assays, which are not only time-consuming but also lack functional target specificity [34,35]. To overcome these limitations and pinpoint the functional constituents, the present study applied a targeted cell-surface affinity approach using living cells as probes to capture functional biological molecules. By using BJ and HMEC-1 as dual biological baits, combined with mass spectrometry identification and background subtraction, we successfully identified GPPVSE, a novel hexapeptide with dual-cell targeting potential, from complex placental extracts. These immortalized cell lines were selected for their robust phenotypic reproducibility to minimize screening variation [36,37]. While they share core reparative pathways with primary human cells, future validation in primary cultures remains warranted to fully reflect complex physiological dynamics [38]. To further ensure capture specificity, the affinity screening was performed at 70–80% cell confluence, because in vitro extracellular matrix (ECM) deposition and structural maturation distinctly lag behind cell proliferation, which effectively minimized non-specific peptide sequestration by the immature matrix [39,40]. Ultimately, by focusing on functional targets on the exposed cell surface, this strategy successfully integrates binding affinity with biological relevance, providing crucial compositional insights for the active reparative constituents within placental extracts.

UniProt sequence alignment revealed that the newly screened peptide GPPVSE is derived from the histone H1.4 and H1.5 precursors. Traditionally, histones function within the nucleus to maintain chromatin structure and regulate gene expression [41]. However, accumulating evidence indicates that they are released into the extracellular space during placental tissue remodeling or cellular stress [42,43]. Subsequently, these extracellular full-length histones are cleaved by microenvironmental proteases into smaller fragments [44,45]. Accordingly, GPPVSE most likely represents a naturally occurring bioactive peptide generated through the extracellular release and proteolytic processing of histones within the placenta. This finding provides a novel molecular basis for the regenerative properties of placental extracts.

In vitro functional assays revealed that GPPVSE exerts stimulatory effects on both fibroblasts and endothelial cells, indicating a dual-acting pro-regenerative profile. Notably, the peptide exhibited a non-linear, bell-shaped dose-response relationship in these assays, with optimal efficacy observed around 20 μM. This biphasic pattern is a recognized hallmark of many receptor-mediated bioactive peptides and growth factors [46]. The reduced efficacy at higher concentrations may result from receptor saturation, subsequent receptor downregulation, or the activation of compensatory negative feedback loops [47,48]. Furthermore, the shared responsiveness of these two cell types suggests that GPPVSE may interact with conserved membrane components. While we hypothesize that ubiquitous surface receptors, such as integrins, heparan sulfate proteoglycans (HSPGs), or G protein-coupled receptors (GPCRs) [49,50,51,52], could potentially mediate this initial cell-surface recognition, these mechanisms are currently speculative. The definitive identity of its membrane receptor(s) and the associated downstream signaling pathways remain experimentally unverified in the present study. Future investigations employing ligand pull-down assays, competitive binding experiments, and specific receptor-blocking strategies are required to strictly define these receptor-ligand interactions.

The in vivo full-thickness skin defect model further validated the pro-regenerative capacity of GPPVSE. Compared with the PBS group, GPPVSE markedly accelerated wound closure. Furthermore, relative to the positive control EGF, it conferred a more comprehensive advantage in the quality of tissue reconstruction, as evidenced by denser granulation tissue, a more structurally integrated epidermal–dermal junction, and pronounced regeneration of hair follicles and sebaceous glands. Wound healing relies on the cooperation of multiple cell types, among which fibroblasts drive extracellular matrix deposition and dermal remodeling, and endothelial cells govern neovascularization and microcirculatory reconstruction [53]. GPPVSE promoted the proliferation and migration of both cell types and further enhanced endothelial tube formation, suggesting that a dual-cell activation effect may coordinately drive dermal matrix remodeling and microvascular network reconstruction in vivo, thereby underpinning the high-quality tissue regeneration observed. Given the ubiquitous involvement of fibroblasts and endothelial cells in diverse tissue repair processes, the dual regulatory effect of GPPVSE points to several potential translational directions worth exploring in future studies, including the management of chronic wounds characterized by impaired angiogenesis and defective matrix synthesis [54], its use as a bioactive ingredient in regenerative cosmeceuticals, and its incorporation as a functional motif into tissue-engineering scaffolds to alleviate vascularization bottlenecks [55].

Nevertheless, several limitations of this proof-of-concept study point to important avenues for our subsequent research. First, regarding the in vivo evaluation, the current therapeutic efficacy was assessed primarily via macroscopic and histological phenotypes using an acute wound model in healthy mice (n = 4 per group). While this cohort size provided sufficient statistical power for initial screening due to the substantial effect size, its regenerative potential must be rigorously validated in larger cohorts under pathological conditions, such as diabetic or chronic ischemic wounds. Concurrently, incorporating specific molecular markers (e.g., CD31 for angiogenesis) will be required to comprehensively map the in vivo mechanisms. Second, from a molecular and structural perspective, while GPPVSE was prioritized based on its dual-cell binding capability and sequence novelty, its specific membrane receptors remain to be elucidated. Future studies utilizing scrambled peptide sequences as negative controls will be essential to rigorously define these receptor–ligand interactions. Furthermore, a dedicated structure–activity relationship (SAR) study utilizing a rationally designed peptide library (including truncated analogs) is necessary to define the absolute minimal functional motif and further optimize its reparative potency. Finally, advancing this peptide toward clinical application requires careful consideration of its dosing and pharmacokinetic profiles. We observed increased inflammatory-cell infiltration at the high concentration of 200 μM, indicating a specific therapeutic window that necessitates careful dose optimization. To establish a comprehensive safety and pharmacokinetic profile, systematic investigations into in vivo peptide stability, metabolic fate, and immunogenicity are essential. Furthermore, developing advanced delivery strategies (e.g., incorporation into sustained-release hydrogels) will be critical to mitigate potential toxicity and degradation, thereby maximizing therapeutic outcomes, particularly in the protease-rich microenvironment of chronic wounds.

## 4. Materials and Methods

### 4.1. Materials and Reagents

The placental extract (lot number: S20230601) was provided by Guizhou Taibang Biological Products Co., Ltd. (Guiyang, China) The peptide (GPPVSE) was synthesized and purchased by China Peptides Co., Ltd. (Shanghai, China). with a purity greater than 98%. The human dermal fibroblasts (BJ) and human microvascular endothelial cells (HMEC-1) were obtained from the Cell Bank of the Chinese Academy of Sciences (Shanghai, China).

### 4.2. Cell Culture

BJ cells were cultured in high-glucose Dulbecco’s Modified Eagle Medium (DMEM) (C11965500BT, Thermo Fisher Scientific, Waltham, MA, USA), and HMEC-1 cells were cultured in Molecular, Cellular, and Development Biology 131 medium (MCDB 131 medium) (10372019, Thermo Fisher Scientific, USA). Both mediums were supplemented with 10% fetal bovine serum (FBS) (A5669701, Thermo Fisher Scientific, USA), −100 U/mL penicillin and 100 μg/mL streptomycin (C3420, VivaCell, Shanghai, China).

All cell types were cultured at 37 °C in a humidified atmosphere containing 5% CO_2_. The culture medium was replaced every other day. Upon reaching 70–80% confluence, the cells were collected and seeded into appropriate culture plates for subsequent experiments.

### 4.3. Peptide Analysis

#### 4.3.1. LC-MS/MS Profiling of Placental Extracts

The placental extract was concentrated under a nitrogen stream and analyzed by LC-MS/MS. Chromatographic separation was performed by reversed-phase chromatography on a Peptide CSH C18 column (1.0 mm × 150 mm, 1.7 μm) (Waters, Milford, MA, USA) by using the UHPLC (Vanquish, Thermo Fisher Scientific, USA). Mobile phases comprised 0.1% (*v*/*v*) formic acid (F112034, Aladdin, Shanghai, China) in water (A) and 60% (*v*/*v*) acetonitrile (A998, Thermo Fisher Scientific, USA) containing 0.1% (*v*/*v*) formic acid (B). The gradient elution procedure was applied as follows: 0–1 min, 3% B; 1–90 min, 3–40% B; 90–120 min, 40–90% B. The flow rate was 0.1 mL/min, the injection volume was 25 µL, and the column temperature was maintained at 60 °C. The column outlet was introduced into an orbitrap mass spectrometer (Exploris 480, Thermo Fisher Scientific, USA) operated in positive electrospray ionization (ESI) mode. The spray voltage was set to 3.5 kV. The capillary and vaporizer temperature were 320 °C and 300 °C, respectively. The sheath gas was 19.8 mL/min. The auxiliary gas was 5 psi. The MS scan range was set to *m*/*z* 500 to 2500, and the resolution was set to 60,000. The RF lens was 45%. The normalized AGC Target was 300%. The maximum ion injection time was 100 ms. The scan event 2 was data-dependent MS/MS and the resolution was set to 15,000. The isolation Window was *m*/*z* 1.6. The maximum ion injection time was 200 ms, the normalized AGC Target was 100%. The MS/MS collision energy was 30%.

#### 4.3.2. Cell-Surface Affinity Screening

To screen for cell-binding peptides, BJ and HMEC-1 cells were cultured in T25 flasks (430639, Corning, Corning, NY, USA) until they reached 70–80% confluence. Cells were washed with phosphate-buffered saline (PBS) and incubated with either placental extracts or PBS as a blank control. The incubation was performed at 4 °C to minimize endocytosis and metabolic internalization, thereby enriching peptides associated primarily with the cell surface. After incubation, uncombined peptides were washed with PBS. The cell-bound peptides were then dissociated using acidic elution buffer, glycine-NaCl, pH 3.0. The final eluate, including a subsequent neutralized PBS rinse, was collected for LC-MS/MS analysis.

#### 4.3.3. LC-MS/MS Analysis of Cell-Bound Peptides

The collected peptide eluate was concentrated under a nitrogen stream and analyzed via LC-MS/MS under the same conditions in Section 4.3.1.

#### 4.3.4. Data Processing and Peptide Identification

All raw MS files were processed using Proteome Discoverer 2.4 software (Thermo Fisher Scientific, USA). Spectra were searched against the UniProtKB *Homo sapiens* database (20,418 sequences, downloaded on 12 February 2025) using the SEQUEST HT search engine (embedded in Proteome Discoverer 2.4). Using no-enzyme search mode and a minimum peptide length of six amino acids. Mass tolerances for precursor and fragment ions were set to 10 ppm and 0.02 Da, respectively. Variable modifications included proline hydroxylation (+15.995 Da), methionine oxidation (+15.995 Da), and N-terminal acetylation (+42.011 Da). The false discovery rate (FDR) was set to 0.01 at both the protein and peptide levels.

### 4.4. Cell Viability Assay

Cell viability was assessed using the Cell Counting Kit-8 (CCK8) (CK001, LABLEAD, Beijing, China) assay. BJ and HMEC-1 cells were seeded into 96-well plates (3599, Corning, USA) at a density of 5 × 10^3^ cells per well in 100 μL of completed medium. Cells were cultured for 12 h to allow attachment. The medium was then replaced with 100 μL of fresh complete medium containing varying concentrations of the peptide—namely, 2, 5, 10, 20, 50, 100, 200, and 500 μM. The untreated group, 0 μM, served as the control group. After 24 h of culture, 10 μL of CCK8 reagent was added to each well. Plates were cultured in the dark for another 2 h. Absorbance at 450 nm was measured using a microplate reader. Cell viability was calculated using the formula [(As − Ab)/(Ac − Ab)], where As, Ac, and Ab represent the absorbance of the peptide-treated sample, the untreated control, and the blank well without cells, respectively.

### 4.5. Cell Proliferation Assay

Cell proliferation was assessed using Trypan blue staining. BJ and HMEC-1 cells were seeded into 48-well plates (3548, Corning, USA) at a density of 2 × 10^4^ cells per well in 200 μL of complete medium. Cells were cultured for 12 h to allow attachment. The medium was then replaced with 200 μL of fresh complete medium containing varying concentrations of the placental peptide, namely 2, 20, and 200 μM. The untreated group, 0 μM, served as the control group. After 24 h of culture, cells were detached from the plates to prepare a cell suspension. An equal volume of Trypan blue solution (C1313, Byotime, Shanghai, China) was added and gently mixed. The number of viable cells was then analyzed using an automatic cell analyzer (Countstar Mira BF, Shanghai Ruiyu Biotech, Shanghai, China). Finally, the relative cell proliferation was calculated by setting the viable cell number of the untreated control group to 1.0.

### 4.6. Cell Scratch Assay

An in vitro cell scratch assay was performed to simulate mechanical skin injury and evaluate cell migration. BJ cells were seeded into 6-well plates (3516, Corning, USA) at a density of 5 × 10^5^ cells per well and cultured for 24 h to form a confluent monolayer. Subsequently, the cells were cultured in low-serum medium (containing 1% FBS) for 8 h. A linear scratch was generated across the center of each well using a sterile 1000 μL pipette tip. Cells were gently washed with PBS to remove non-adherent cellular debris. The cells were then cultured in a low-serum medium containing 1% FBS and different concentrations of the peptide: 2, 20, and 200 μM. The 0 μM group served as the control. Images were acquired at fixed fields of view at 0, 16, and 40 h after scratching using an inverted microscope (Eclipse-Ti, Nikon, Tokyo, Japan). The remaining wound areas were quantified using ImageJ 1.54g software (NIH, Bethesda, MD, USA). The wound closure rate was calculated using the following formula: [(A0 − At)/A0], where A0 represents the initial scratch area at 0 h, and At represents the remaining scratch area at 16 or 40 h.

### 4.7. Transwell Migration Assay

A Transwell assay was used to evaluate the effect of the peptide on HMEC-1 migration. HMEC-1 cells were resuspended in a low-serum medium (1% FBS). A total of 200 μL of the cell suspension containing 1 × 10^4^ cells was added into the upper chamber of a Transwell (3464, Corning, USA) insert with an 8.0 μm pore size. The lower chamber was filled with 600 μL of complete medium supplemented with the peptide at 2, 20, or 200 μM. The 0 μM group served as the control group. After 8 h of culture, the inserts were removed, and the non-migrated cells on the upper surface of the membrane were gently removed using a cotton swab. Migrated cells on the lower surface were then fixed with 4% paraformaldehyde and stained with 0.1% crystal violet. Three randomly selected fields from each insert were photographed using an inverted microscope (Thunder Imager, Leica, Wetzlar, Germany), and the number of migrated cells was quantified using ImageJ 1.54g software.

### 4.8. Tube Formation Assay

An in vitro tube formation assay was performed to evaluate the pro-angiogenic activity of the peptide. Matrigel (356234, Corning, USA) was added to a 96-well plate at 20 μL per well and allowed to polymerize at 37 °C for 2 h. HMEC-1 cells were resuspended in complete medium containing the peptide at 2, 20, or 200 μM. The 0 μM group served as the control. A total of 100 μL of the cell suspension containing 1 × 10^4^ cells was added to each well and cultured for 8 h. Randomly selected fields were photographed using an inverted microscope (Thunder Imager, Leica, Germany). Total tube length and junction number were quantified using the Angiogenesis Analyzer plugin for ImageJ 1.54g software.

### 4.9. Animal Study

The C57BL/6 mice (male, 4–5 weeks) were purchased from Beijing Viewsolid Biotechnology Co., Ltd. (Beijing, China) All animals were bred in the pathogen-free facility with a 12 h light/dark cycle at temperature (20–25 °C), relative humidity (40–60%), and had ad libitum access to food and water. All animal experiments in this article were carried out in accordance with guidelines evaluated and approved by the Ethics Committee (IPEAECA2026063).

### 4.10. In Vivo Wound Healing of Peptide

A full-thickness skin wound approximately 6 mm in diameter was created on the dorsal region of each mouse. The mice were then randomly allocated into four groups (n = 4 per group) using a computer-generated randomization sequence and received topical applications of the respective solutions twice daily: PBS (vehicle control), 20 μM EGF (positive control) (E9644, Merck KGaA, Darmstadt, Germany), 20 μM peptide, and 200 μM peptide. Wounds were photographed on days 0, 4, 8, and 12 with a reference scale bar included in the field of view. The wound areas were quantified using ImageJ 1.54g software, and all measurements were calibrated against the scale bar. To eliminate observer bias, all wound-area measurements and subsequent histological evaluations were performed by investigators strictly blinded to the treatment group assignments.

### 4.11. Histological Analysis

On days 8 and 12, the mice were euthanized, and the regenerated skin samples were harvested. The tissue samples were fixed in 4% paraformaldehyde (PFA), embedded in paraffin, and sectioned. The sections were then subjected to hematoxylin and eosin (H&E) staining. Histological images were captured using an inverted microscope (Thunder Imager, Leica, Wetzlar, Germany). For the quantitative evaluation of histological features, the thickness of the neo-epithelium was measured on a representative section from each group using ImageJ 1.54g software. Briefly, ten distinct locations were randomly selected along the regenerated epithelium within the representative section, and the vertical thickness was measured to objectively reflect the local epidermal uniformity and thickness.

### 4.12. Statistical Analysis

All data are expressed as the mean ± standard deviation (SD). Statistical analysis and graphing were performed using Origin 2021 software (OriginLab, Northampton, MA, USA). Differences between two groups were evaluated using an independent samples *t*-test. Comparisons among three or more groups were performed using one-way analysis of variance (ANOVA) followed by Tukey’s post hoc test. A *p* < 0.05 was considered statistically significant.

## 5. Conclusions

In summary, by employing the cell-surface-targeted screening strategy, this study successfully discovered and identified GPPVSE, a novel peptide derived from placental extracts. We revealed its natural biological origin from histones H1.4/H1.5 and validated its significant pro-regenerative effects on skin repair both in vitro and in vivo. This work not only provides preliminary molecular insights for the reparative functions of placental extracts, but also suggests a potential foundation for the development of placental peptide-based biologics for skin repair.

## Figures and Tables

**Figure 1:**
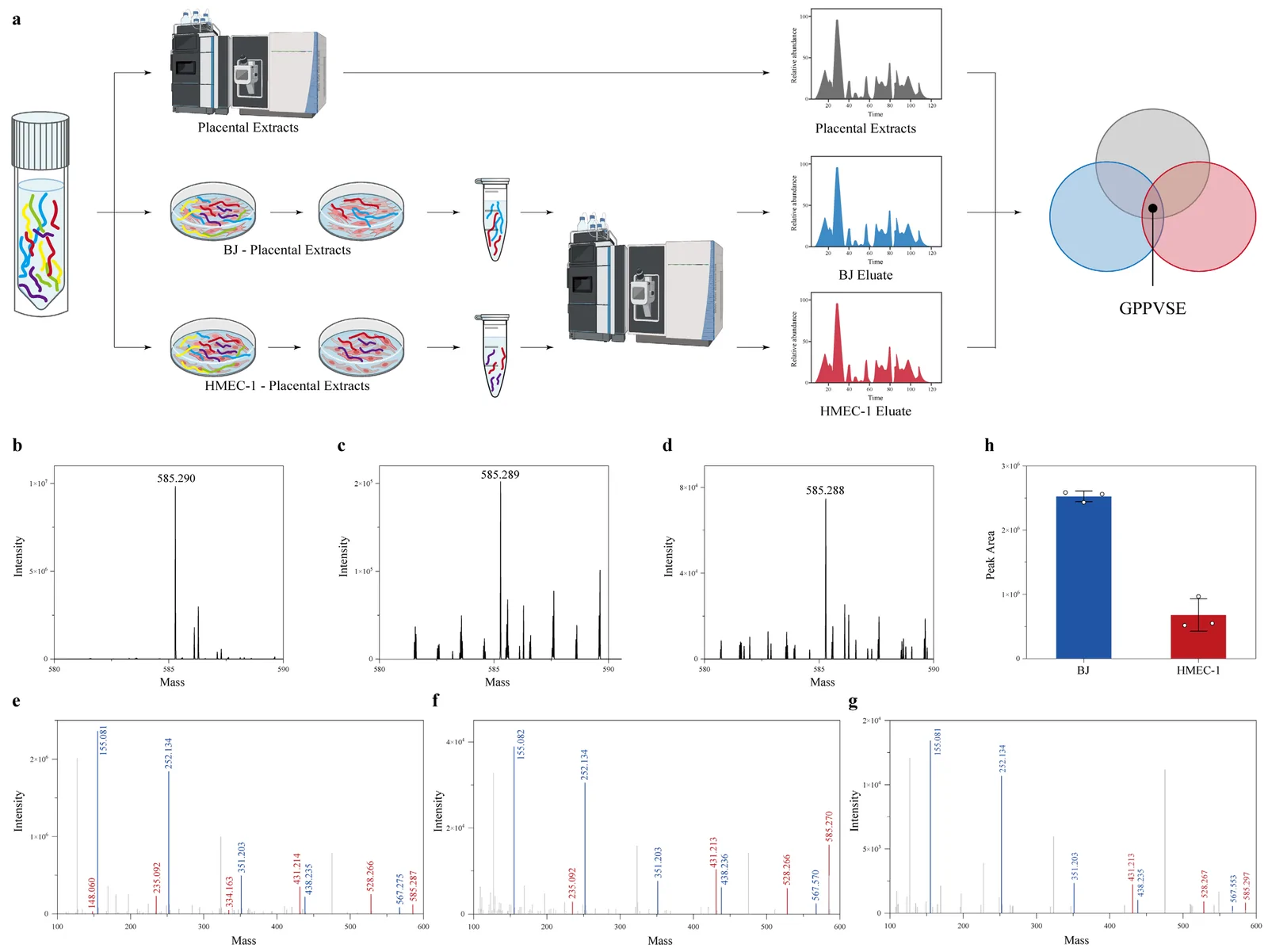
Mass spectrometric identification of the cell-binding placental peptide. (**a**) Diagrammatic sketch of cell surface affinity screening analysis; (**b**–**d**) MS1 spectrum of peptide GPPVSE in the placental extract (**b**), BJ eluate (**c**), and HMEC-1 eluate (**d**); (**e**–**g**) MS/MS spectrum of peptide GPPVSE in the placental extract (**e**), BJ eluate (**f**), and HMEC-1 eluate (**g**); (**h**) extracted ion chromatogram peak areas of peptide GPPVSE recovered from BJ and HMEC-1 cells; data are presented as Mean  ±  SD (n = 3).

**Figure 2:**
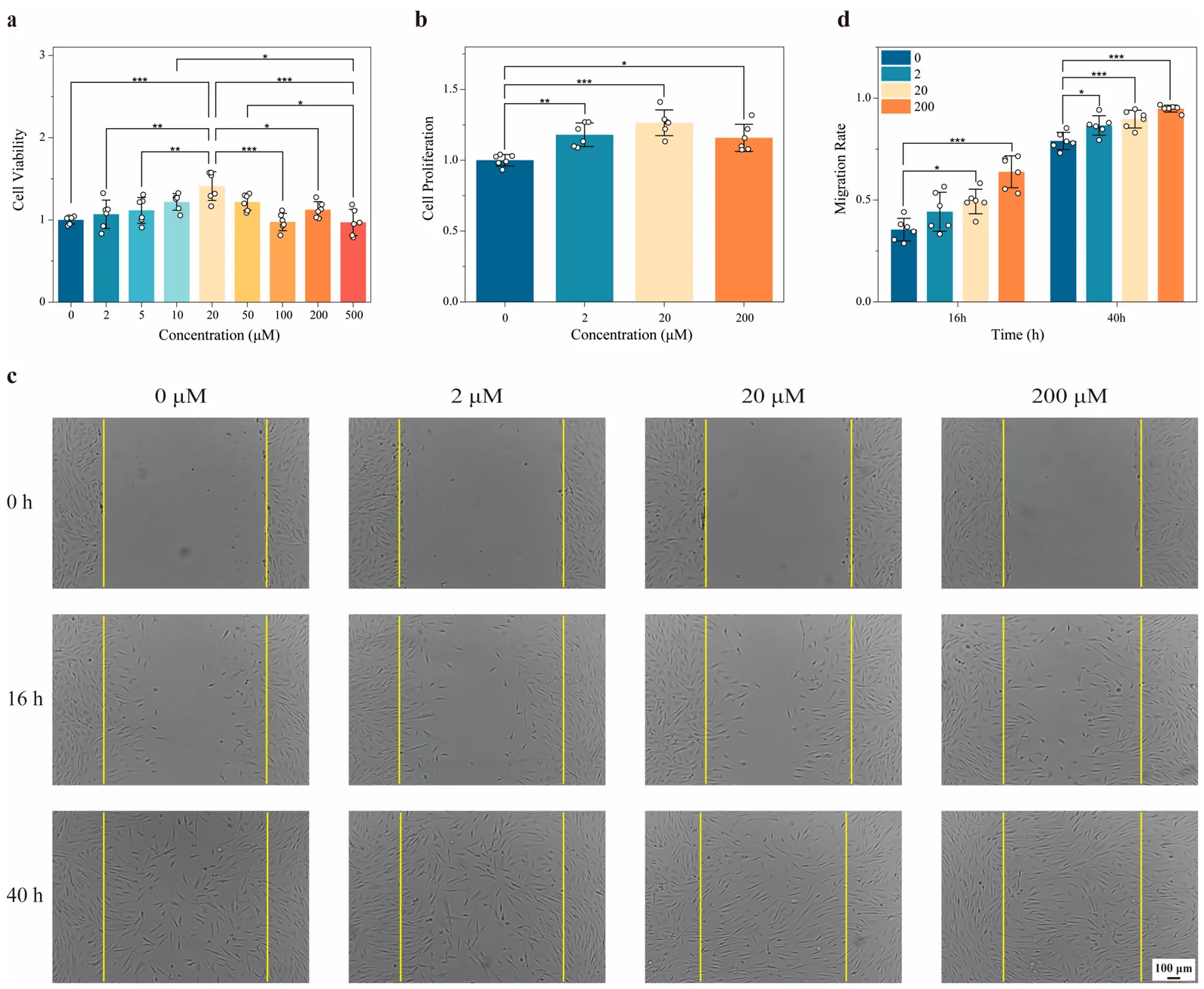
The effect of the peptide on the viability, proliferation and migration of fibroblasts in vitro. (**a**) Relative cell viability of BJ treated with the peptide for 24 h; (**b**) relative cell proliferation of BJ treated with the peptide for 24 h; (**c**) representative images of the scratch wound healing assay in BJ treated with the peptide at indicated time points (scale bar = 100 μm); (**d**) quantitative analysis of the wound closure rates at the corresponding time points; all data are expressed as the mean ± SD (n = 6), * *p* < 0.05, ** *p* < 0.01, and *** *p* < 0.001. The white dots represent individual data points, and the yellow solid lines indicate the initial boundaries of the scratch wound.

**Figure 3:**
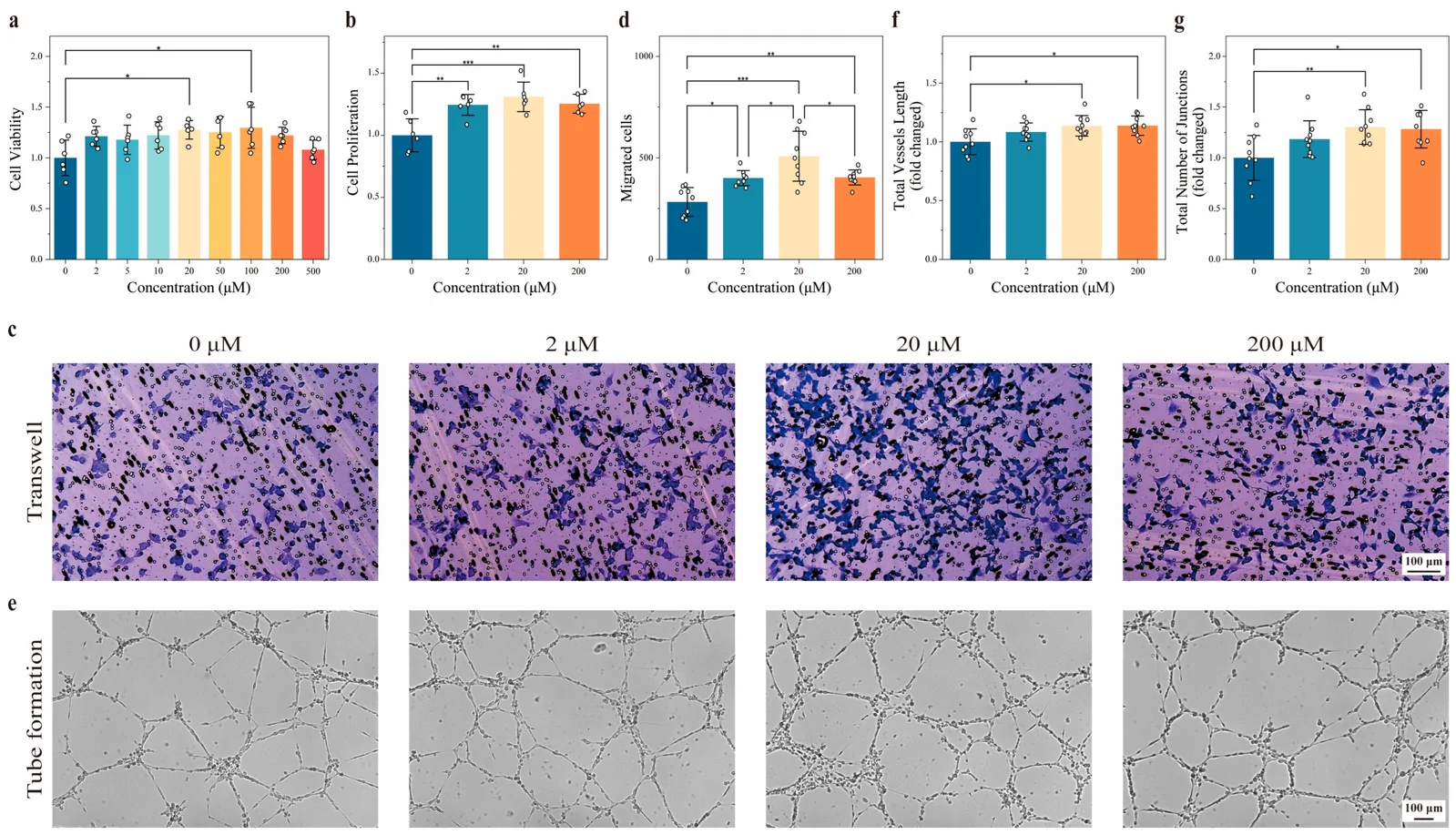
The effect of peptide on the viability, proliferation, migration and pro-angiogenic activity of microvascular endothelial cells. (**a**) Relative cell viability of HMEC-1 treated with the peptide for 24 h; (**b**) relative cell proliferation of HMEC-1 treated with the peptide for 24 h; (**c**) representative images of the Transwell migration assay in HMEC-1 (scale bar = 100 μm); (**d**) quantitative analysis of migrated HMEC-1; (**e**) representative images of Matrigel tube formation by HMEC-1 (scale bar = 100 μm); (**f**,**g**) quantitative analysis of the total vessel length (**f**) and total number of junctions (**g**); all data are expressed as the mean ± SD (n = 6 for (**a**,**b**); n = 9 for (**d**,**f**,**g**)), * *p* < 0.05, ** *p* < 0.01, and *** *p* < 0.001. The white dots represent individual data points.

**Figure 4:**
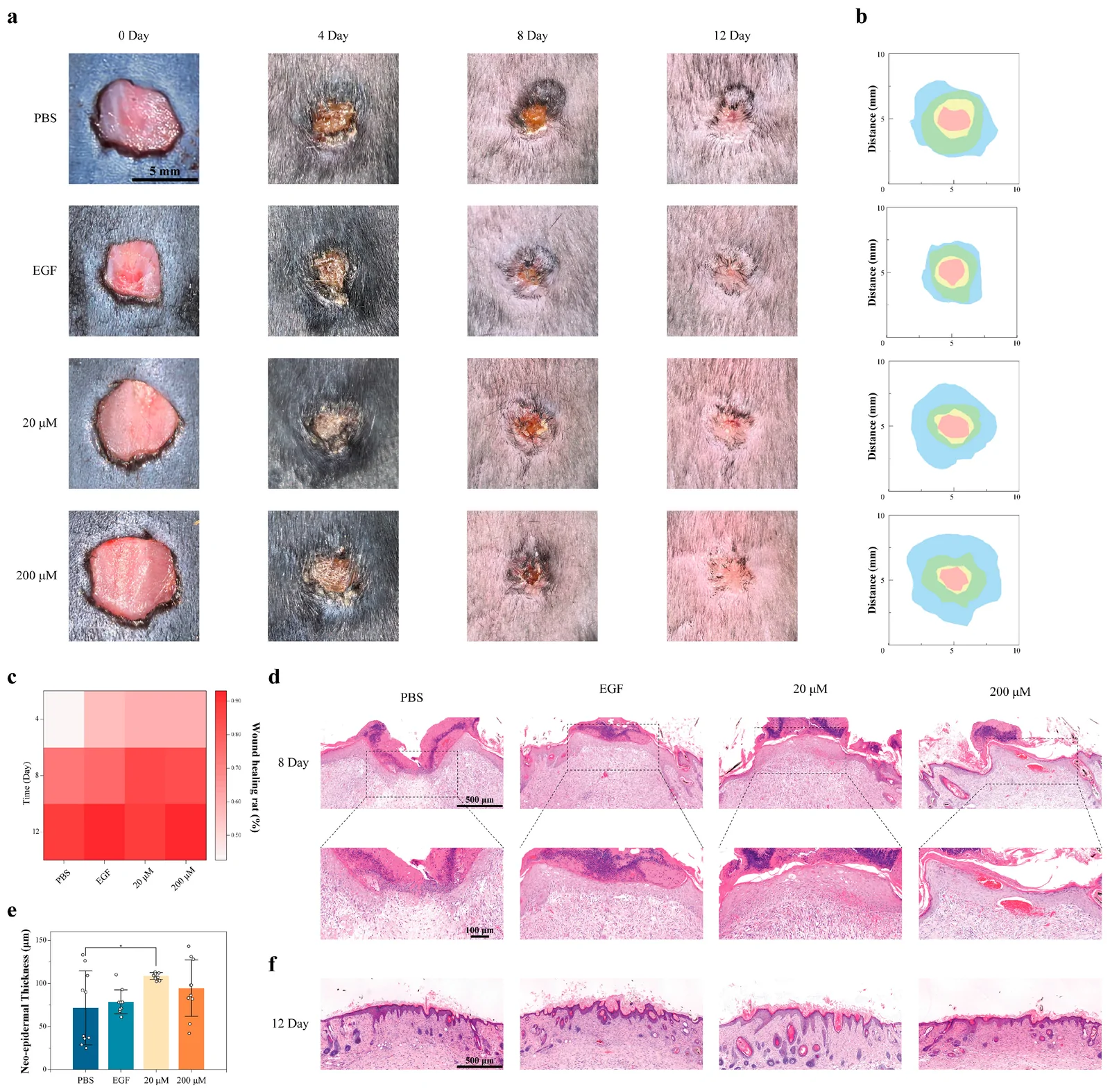
Effects of the peptide on promoting wound healing. (**a**) Representative photographs of the wounds in different treatment groups (scale bar  =  5 mm); (**b**) superimposed traces of wound areas under various treatments on days 0 (blue), 4 (green), 8 (yellow), and 12 (pink); (**c**) wound healing rate within 12 days; (**d**) H&E staining of wound tissue with different treatments on the 8th day (scale bar  =  500 μm); (**e**) neo-epidermal thickness of different treatments on the 8th day; data are expressed as the mean ± SD; n = 10 measurement points per representative section, * *p* < 0.05, the white dots represent individual data points.; (**f**) H&E staining of wound tissue with different treatments on the 12th day (scale bar  =  500 μm).

**Table 1:** Details of the peptides identified in the placental extract.

No.	Peptide Sequence	MW (Da)	Xcorr	Abundance
1	MEVKPPP	839.433	2.82	1.60 × 10^6^
2	MEVKPPPG	896.455	2.56	1.53 × 10^6^
3	GPPVSEL	698.372	2.39	5.75 × 10^5^
4	MPIPPPPP	714.418	2.24	5.86 × 10^5^
5	MLLLLL	584.438	2.23	5.77 × 10^5^
6	LPQVPISGL	923.556	2.22	6.77 × 10^5^
7	IPPPPPP	714.418	2.21	5.86 × 10^5^
8	LPPGLAVKE	923.556	2.12	6.77 × 10^5^
9	GPPVSELITK	1040.599	2.06	4.90 × 10^5^
10	GPPVSE	585.288	2.03	1.53 × 10^7^
11	MSDKPDM	734.303	1.95	1.49 × 10^6^
12	VPPPPP	603.350	1.81	4.90 × 10^5^
13	MADKPDM	718.308	1.57	4.60 × 10^5^
14	IGPVGA	513.303	1.54	4.55 × 10^5^
15	GLPVGA	513.303	1.52	4.55 × 10^5^
16	AVPVGA	513.303	1.52	4.55 × 10^5^
17	VPAVGA	513.303	1.47	4.55 × 10^5^
18	MAAGGPGAG	557.268	1.37	5.35 × 10^5^
19	GTFVSP	607.309	1.27	4.20 × 10^5^
20	TFGVSP	607.309	1.27	4.20 × 10^5^

**Table 2:** Mass spectrometric parameters for identification of the peptide GPPVSE.

Sample	Precursor *m*/*z*	Charge	Theoretical Mass	Mass Error	RT	XCorr
Placental extracts	585.290	1	585.288	2.90 ppm	2.28 min	2.03
BJ eluate	585.289	1	585.288	1.54 ppm	2.37 min	1.85
HMEC-1 eluate	585.288	1	585.288	0.51 ppm	2.25 min	1.43

## Data Availability

The original contributions presented in this study are included in the article. Further inquiries can be directed to the corresponding author.

## Supplementary Materials

The following supporting information can be downloaded at https://www.mdpi.com/article/10.3390/ijms27198617/s1.

## Abbreviations

The following abbreviations are used in this manuscript:

BJ	human dermal fibroblasts
HMEC-1	human microvascular endothelial cells
PBS	phosphate-buffered saline
EGF	epidermal growth factor
FBS	fetal bovine serum
LC-MS/MS	liquid chromatography–tandem mass spectrometry
CCK8	Cell Counting Kit-8
SD	standard deviation
H&E	hematoxylin and eosin
