# Supplementary material for: Identification of a Novel Placental Peptide with Skin Reparative Properties by Cell-Surface Affinity Screening
Source: Int J Mol Sci. 2026 Sep 26;27(19):8617. doi: 10.3390/ijms27198617 (PMC13665333; doi:10.3390/ijms27198617)
Supplement: Supplementary file 1 [file ijms-27-08617-s001.zip › ijms-4567602-supplementary.pdf]

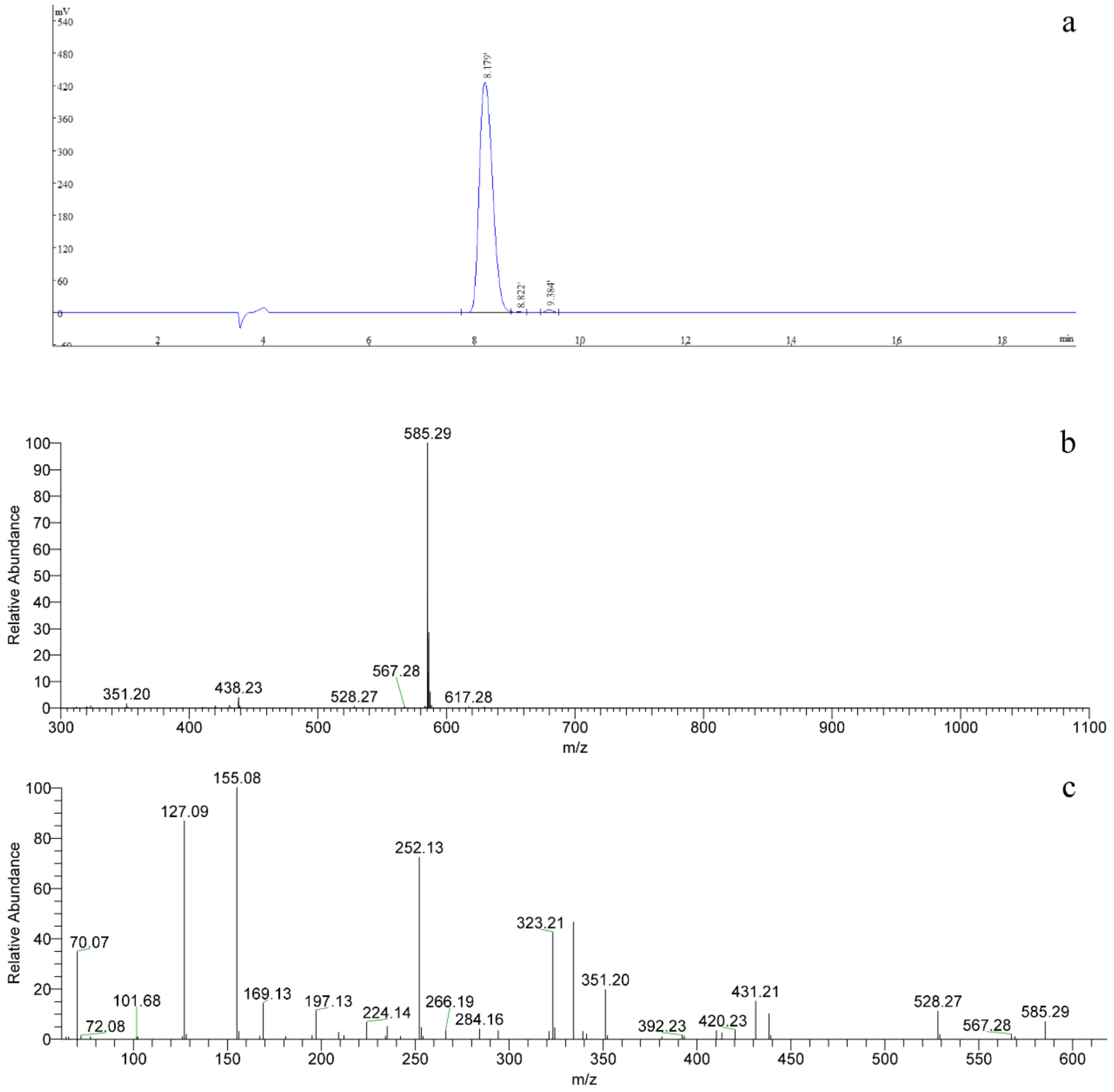

Figure S1. Characterization of the synthetic peptide GPPVSE. (**a**) High-Performance Liquid Chromatography (HPLC) chromatogram of the synthetic peptide. The single major peak indicates a high purity of >98%. (**b**) Full scan mass spectrum (MS) of the synthetic peptide, showing the primary ion peak corresponding to its theoretical mass: *m/z* 585.29 [M+H]+. (**c**) Tandem mass spectrum (MS/MS) of the synthetic peptide. The fragment ions confirm the exact amino acid sequence of GPPVSE.
